# Synthetic Cannabinoids (SCs) K2 clinical manifestation-A literature review

**DOI:** 10.1192/j.eurpsy.2023.1003

**Published:** 2023-07-19

**Authors:** S. Kamrun, T. Sultana, F. A. Faruki, S. Goddu

**Affiliations:** 1 Harlem Hospital Center; 2 Research Unit, Manhattan Psychiatric Center, NY; 3 Psychiatry Dept., Bergen New Bridge Medical Center, Paramus, NJ; 4 Psychiatry Dept., Harlem Hospital Center, NY, United States

## Abstract

**Introduction:**

There has been an increase in the use of new psychoactive substances containing synthetic cannabinoids in recent years. It is also known as K2, spice, or fake weed, and these are popular as recreational drugs [Kourouni I et al. JAMA NetW Open 2020 Jul; 1;3(7): e208516]. CB1 agonists in SCs mimic the effects of cannabis, making users feel happy and relaxed. However, recreational SCs may result in unwanted severe consequences such as acute anxiety and psychosis [Gunderson EW et al. AM J Addict 2012 Jul-Aug; 21(4): 320-6]. Relative to tetra-hydro cannabinol (THC), synthetic cannabinoids (SCs) are more potent and efficacious agonists and may exert deleterious effects on health [Gunderson EW et al. AM J Addict 2012 Jul-Aug; 21(4): 320-6].

**Objectives:**

Our aim of this review is to focus on the typical presentation of SCs use and help clinicians to better understand and be more vigilant about K2 manifestations

**Methods:**

In conducting the literature review, only English language articles were selected from PubMed and PubMed Central (PMC) databases through August 14, 2022, using the search term “Synthetic cannabinoids k2 clinical manifestation”. Three reviewers conducted the initial review of the titles and abstracts of the electronic search, followed by detailed assessments of the relevant studies. Peer-reviewed Case series, case reports, and systematic review studies were included that met the inclusion criteria (articles in the English language, studies on humans, studies on synthetic cannabinoids (SC) or K2 use and its clinical manifestations.

**Results:**

Electronic search results showed a total of 60 articles. Fifty articles were excluded based on title review (36 articles), abstract review (4 articles), and full article review (10 articles). Case series on 30 ICU patients (SC intoxication) showed agitation 33%, bizarre behavior, coma 33%, seizure 20%, Acute respiratory failure 60%, Tracheal intubation 70%, Rhabdomyolysis 26%, Invasive mechanical ventilation 40%, Acute kidney injury 26% and cardiotoxic effect [Kourouni I et al. JAMA NetW Open 2020 Jul; 1; 3(7): e208516]. Cross-sectional study on 50 male cases showed altered perception 68%, including auditory and visual hallucination, dizziness and loss of consciousness, palpitation 76%, chest pain 12%, panic attack, and convulsion [Abdelmoneim WM et al. Middle East Current Psychiatry 2022; 29 (1): 24]. Literature reviews has shown the common psychiatric presentations of SC are acute anxiety, agitation, psychosis, paranoia, disorientation, alteration of mood and perception, hallucination, and delusion [Debruyne D et al. Subst Abuse Rehabil. 2015 Oct 20; 6:113-29, Radhakrishnan R et al. Front Psychiatry 2014 May 22;5:54].

**Conclusions:**

There are limited research related to synthetic cannabinoids in human. Based on our review, SC intoxication can be life-threatening besides psychiatric manifestation. Therefore, clinicians must understand the vast clinical manifestations of SCs.

**Disclosure of Interest:**

None Declared

